# Lotus-fish co-culture reshapes pond microbiota and improves ecological stability relative to fish monoculture

**DOI:** 10.3389/fmicb.2026.1922152

**Published:** 2026-08-26

**Authors:** Shandong Chen, Yan Miao, Jiayu Yin, Zexun Zhou, Ye Yuan, YunYun Liu, Yongchun Li, Lei Zeng, Chongqing Wang, Zhongyuan Shen, Wuhui Li

**Affiliations:** 1 Engineering Research Center of Polyploid Fish Reproduction and Breeding of the State Education Ministry, College of Life Sciences, Hunan Normal University, Changsha, China; 2 Hunan Yuelu Mountain Science and Technology Co., Ltd. amid at Aquatic Breeding, Changsha, China; 3 Hunan Buckwheat Lake High Quality Fish Research Institute Co., Ltd., Yueyang, China

**Keywords:** algae community, bacterial community, fish growth indicators, lotus-fish co-culture, sediments organic matter components

## Abstract

**Introduction:**

The lotus-fish co-culture ponds (LP) is a widely applied integrated aquaculture system in China. However, research on the micro-ecology of aquaculture in lotus ponds is still insufficient. This study explored the dynamic patterns of organic matter composition, as well as algal and bacterial community structures in LP and MP.

**Methods:**

A total of six independent ponds were used in this study: three lotus ponds designated as the lotus-fish co-culture ponds group (A1, A2, A3), and the other three conventional earthen ponds serving as the fish monoculture ponds group (B1, B2, B3). Bacterial and algae community survey, environment factor survey, bacterial Biomarker selection, function prediction, and association analysis were used to analyze the effects of lotus-fish co-culture on algae, bacteria and sediment organic matter components of pond.

**Results:**

The sediments of LP had lower concentrations of total nitrogen (TN), total phosphorus (TP), total carbon (TC) and organic matter (OM), and the chlorophyll*-a* (chl*a*) content in the water was also relatively low. The algal community in LP exhibited seasonal variations, while MP was predominated by *Chlorophyta* and *Cyanobacteriophyta*. The dominant bacterial phyla were the same in both pond groups, yet their abundances varied across months. Bacterial biomarkers in LP was *c_Alphaproteobacteria*, *c_Vicinamibacteria*, etc. (sediments) and *c_Gammaproteobacteria* (water), whereas MP was *c_Desulfobacteri*a, *c_Dehalococcoidia*, etc. (sediments), and *c_Verrucomicrobiia*, *c_Mycobacteriales*, etc. (water). Functional analysis suggested that bacterial communities in LP were enriched in methanotrophy, nitrogen fixation, whereas MP communities were more closely associated with anaerobic respiration and phototrophic processes. The bacterial network in LP exhibited a modular structure reliant on keystone taxa, whereas MP harbored highly interconnected and cooperative bacterial communities. In the LP sediments, TC, TN, and TP collectively drive the broad differentiation of bacterial modules. Total nitrogen (TN) played a vital role in bacterial assembly in MP sediments, while total phosphorus (TP) acted as the primary negative driving factor in MP sediments. FBL and FBW were significantly greater in LP than MP, while CF was lower.

**Discussion:**

These findings indicate that microbial communities in LP and MP follow distinct assembly patterns, and they differ in core ecological functions and environmental preferences within their respective ecosystems.

## 1 Introduction

Currently, about 47.98% of aquaculture products come from pond farming in China (Liu et al., 2020). High-productivity, high-density pond farming has led to organic pollutant discharge, causing water pollution, and fish quality deterioration (Ottinger et al., 2016; Hu et al., 2018). Co-culture system, such as rice-fish and lotus-fish, has been evaluated as one of the most efficient and environmentally sustainable methods of the 21st century (FAO, 2014; Okomoda et al., 2023). Co-culture system combines hydroponic of plants, aquaculture of fish and chemical filtration of microorganisms to form an ecological cycle where fish fertilize the water, plants purify it, and the water sustains the fish. Co-culture system systems offer numerous benefits compared to fish monoculture systems, such as faster growth rates, higher yields, and lower environmental impact with less energy consumption. For example, pumpkin-catfish co-culture system significantly increased fish productivity and survival, purified water quality and significantly reduced NO^2–^ levels (Oladimeji et al., 2020). The rice-fish co-culture system enhanced soil bacterial complexity, stability, and resistance, improved organic matter decomposition, and promoted plant growth over fish monoculture (Noppol et al., 2022; Hou et al., 2024). Currently, Co-culture system has limited system types and modes, and efficient aquaculture-plant pairings are rarely studied.

As an ecological co-culture system, introducing fish into lotus ponds prevents excessive competition among fish and reduces the risk of fish disease outbreaks. Fish stocking optimizes material and energy cycles within the lotus ponds ecosystem, enabling resource sharing and mutual benefits. Lotus-fish co-culture reduces inputs of chemical fertilizers, pesticides, and feed, lowering cultivation and aquaculture costs while delivering low-carbon ecological benefits. Lotus culture provides fish with natural food sources and enriches the trophic structure (Li et al., 2023). In lotus ponds, fish can feed on natural food sources such as fallen lotus, aquatic insects, and plankton. Fish activity reduces pest infestations on lotus plants, improves aeration of the bottom sediments, and promotes lotus root growth (Ma et al., 2017). Lotus effectively purifies nutrients in water and sediments by absorbing carbon, nitrogen, phosphorus, and organic matter from fish feces and sediments, while simultaneously reducing nutrient loss at the sediments-water interface (Chao et al., 2019; Yang et al., 2024). And lotus reduce the abundance of algae and aerobic bacteria by competing with algae for sunlight and nutrients and with aerobic bacteria for organic matter (Yang et al., 2022b). As a result, the lotus plants purify the water and increase oxygenation, providing healthy growing environments for fish.

Sediments and water microorganisms are key components of the aquaculture ecosystem, collectively participating in material cycling, energy flow, and nutrient transformation within the ponds (Yang et al., 2014). Analyzing the distribution of microorganisms in water and sediments, Li et al. found that bacteria predominated in both abundance and diversity, with organic matter-degrading taxa dominating the sediments and phototrophic taxa abundant in the water (Dai et al., 2021). Simultaneously, these bacteria, both probiotic and pathogenic, are essential to the survival environment of aquaculture fish and to the constitution of micro-ecosystems (skin, gut, etc.). In an co-culture system, probiotics can be enriched in the fish gut to promote energy metabolism, strengthen host immunity and improve gut health (Padmavathi et al., 2012; Wongkiew et al., 2018). In addition, the presence of pathogenic bacteria and parasites in the sediments is harmful to fish health and is a major source of fish pathogens. Much research has been conducted on the effects of rice-fish co-culture ponds on micro-ecology, but little is known about the microbial community structure and function in sediments and water of extensive lotus-fish co-culture ponds. In this study, grass carp (*Ctenopharyngodon Idella)* was cultured in lotus ponds and mud ponds. Water and sediments samples were collected bimonthly from September 2023 to July 2024. To thoroughly investigate the differences in micro-ecology between lotus-fish co-culture ponds and fish monoculture ponds, this study systematically compared and analyzed the organic matter components, bacterial metabolic activity, and microbial community diversity and composition in both water and sediments. The research findings provide preliminary research data for lotus-fish co-culture.

## 2 Materials and methods

### 2.1 Experimental setup

All the experimental procedures were conducted following the standards and ethical guidelines established by the Animal Ethical Review Committee of Hunan Normal University, Changsha, China. Experiments were established in six 150 mu ponds at the Hunan Buckwheat Lake High Quality Fish Research Institute, Yueyang City, Hunan Province, China. A total of six independent ponds were used in this study: Three ponds designated as lotus-fish co-culture ponds (LP) (A1, A2, and A3) were planted with 500 kg of lotus roots per mu in each pond, and the other three conventional earthen ponds serving as the fish monoculture ponds (MP) group (B1, B2, B3). Each ponds covered an area of 150 mu, and the water depth was maintained steadily at 1.0–1.8 m. Louts (*Nelumbo nucifera Gaertn*) was cultivated in ponds A1, A2, and A3 to establish the lotus-fish co-culture ecological aquaculture model, while no lotus was planted in ponds B1, B2, and B3, which were set as the control group. All ponds were supplied with natural rainwater and water from surrounding agricultural ditches, with identical hydrological and topographical conditions across sites. Before stocking, grass carp were uniformly acclimated. Individuals with poor health, injuries or abnormal body size were excluded to ensure consistent initial health status and body specifications among all groups. No herbicides or pesticides were applied throughout the experiment in the lotus ponds. The experimental grass carp were artificially reared healthy individuals with an initial body weight of (75 ± 8.23) g (mean ± SD). Fish stocking was completed in July 2023, with 75,000 individuals released into each ponds, corresponding to a uniform rearing density of 500 fish per mu. During the culture period, 100 kg compound feed (containing ≥ 30.0% crude protein, ≥ 5.0% crude fat, ≤ 15.0% crude ash, ≤ 12.0% crude fiber, ≥ 1.6% lysine, ≥ 1.0% total phosphorus, and ≤ 12.0% moisture per kilogram of feed) was supplied each ponds at fixed times every day. No additional aeration equipment was used throughout the trial to simulate natural ponds aquaculture conditions. Sample collection was carried out from September 2023 to July 2024 at 2-month intervals. Sample collection was conducted from September 2023 to July 2024 at 2-month intervals. The five-point sampling method was applied to all ponds, with sampling sites set at the center and four corners of each pond. Five subsamples collected from different locations within the same pond were fully mixed to minimize sampling errors caused by spatial heterogeneity. Water samples (500 mL each) were collected at a depth of 0.3–0.5 m below the water surface at five sampling points in each pond using a glass water sampler. Surface sediment samples (50 g each) were collected from the top 0 to 0.1 m layer at the bottom of five sampling points per pond with a sediment sampler. All samples were immediately transported to the laboratory and stored at 4 °C. After preprocessing including centrifugation and filtration, the samples were preserved in an ultra-low temperature freezer at −80 °C for subsequent analysis. The abbreviations of samples are defined as follows: LM = sediment samples from lotus ponds (LP); LW = water samples from lotus ponds (LP); MM = sediment samples from monoculture ponds (MP); MW = water samples from monoculture ponds (MP).

Fish were measured and weighed at three time points: the start of the experiment, 6 months and 1 year. Twelve fish were randomly sampled from each pond groups to record body weight and length. Calculation formulas for condition factor (CF), weight gain rate (WGR) and specific growth rate (SGR) are shown below:


SGR=([ln(finalweight)-ln(initialweighjt)

Numberofexperimentdays)×100

WGR=[(final⁢weight-initial⁢weight)/initial⁢weight]×100

$$\mathrm{CF}=\mathrm{final}\,\mathrm{weight}/\mathrm{final}\,{\mathrm{length}}^{3}$$

### 2.2 Algae, chlorophyll *a* sampling and statistics

Algae sampling, counting and species identification were conducted according to previous studies and the Technical Code for algae Monitoring in Inland Waters (SL 733-2016) issued by the Ministry of Water Resources of People’s Republic of China (Tie et al., 2018). Qualitative samples were collected using a No. 25 plankton net at 0–0.5 m depth with a “∞”-shaped tow for 3–5 min, fixed with Lugol’s solution, stored in the dark at 4–10 °C for up to 48 h, and observed microscopically to identify algae to genus or species. Quantitative samples (1–2 L) were collected at 0.5 m depth, fixed with Lugol’s solution, settled for 48 h in the lab, supernatant siphoned off, sediments adjusted to 30 mL, and counted in a 0.1 mL counting chamber under 40–600 × magnification using the row method (2nd, 5th, 8th rows), then converted to cells per liter. The formula for counting the number of algae in 1 L water sample is as follows:


$$N=\frac{N_{0}}{N_{1}}\times \frac{V_{1}}{V_{0}}\times P_{\text{n}}$$

N: Number of algae in 1 L water sample, cell/L; N_0_: Total number of squares; N_1_: Number of squares counted; V_1_: Volume of 1 L water sample after concentration, mL; V_0_: Volume of counting frame, mL; P_*n*_: Number of algae counted.

Chlorophyll *a* in the water samples was extracted by the hot ethanol method and chlorophyll *a* in the samples was determined by colorimetry (Yuwei et al., 2006). Samples were extracted with ∼12 mL of 90% ethanol in a 250 mL flask, preheated in an 80–85 °C water bath, followed by addition of 4 mL hot ethanol and heating for 2 min. The mixture was kept in the dark at room temperature for 4–6 h ( ≤ 12 h), then filtered through a 25 mm glass fiber membrane and adjusted to 10 mL. Absorbance was measured at 66 and 750 nm with 90% ethanol as reference (E665, E750). After adding one drop of 1 mol/L HCl, the sample was shaken, left for 1 min, and remeasured at 665 and 750 nm (A665, A750). Chlorophyll *a* is calculated as follows:


$$C\,h\,l\,a=\frac{27.9\,V_{C_{2}\,H_{10}\,O\,H}\,\left[\left[E_{665}-E_{750}\right]-\left(A_{665}-A_{750}\right)\right]}{V_{S\,a\,m\,p\,l\,e}}$$

Chl*a* denotes chlorophyll *a* concentration (mg/m^3^). V_*C_2H_5OH*_ represents the volume of solvent used for extraction (mL), while V_*sample*_ denotes the volume of filtered water sample.

### 2.3 Determination of organic matter components in ponds sediments

Total carbon (TC) was determined using an elemental analyzer (Sercon Integra 2, United Kingdom) via high temperature combustion. Total nitrogen (TN) was determined by the automatic Kjeldahl method (NY/T 1121.24-2012). The total phosphorus (TP) was determined by the Olsen method, following the reference “*Soil and Agricultural Chemistry Analysis*.” Organic matter (OM) was determined by the potassium dichromate oxidation method (NY/T 1126.6-2006) (Bao, 2000).

Total nitrogen (TN) was determined via Kjeldahl digestion-distillation titration (NY/T 1121.24-2012). Briefly, 0.5000 g sediment was digested with mixed catalyst and concentrated H_2_SO_4_ at 420 °C for 2 h. Ammonia was distilled and absorbed by boric acid-indicator solution, titrated with 0.01 mol/L HCl to calculate TN content (g/kg).

Total carbon (TC) was measured by dry combustion elemental analysis (Sercon Integra 2). Homogenized 100-mesh powder was sealed in tin capsules, combusted at 1,000 °C, reduced at 600 °C, and quantified against δ^13^C V-PDB reference material.

Total phosphorus (TP) was determined by alkali fusion–molybdenum-antimony-ascorbic acid spectrophotometry. A 0.2 g subsample was fused with NaOH in a muffle furnace (640 °C, 15 min); extracts were centrifuged, diluted, and colored for 30 min. Absorbance was read at 700 nm on a UV–vis spectrophotometer, and TP (g/kg) was calculated from external calibration curve.

Organic matter (OM) was quantified by dichromate wet oxidation titration. After oil-bath oxidation at 170–180 °C for 5 min, residual dichromate was titrated with ammonium ferrous sulfate. A correction factor of 1.1 (oxidation efficiency) and Van Bemmelen factor 1.724 were applied to convert organic carbon to OM (g/kg).

### 2.4 Bacterial DNA extraction and high-throughput sequencing

Prior to DNA extraction from water samples, one liter of composite water sample was collected from each of the six ponds, yielding six water samples in total. The water was filtered through 0.22 μm GF/C membranes to trap aquatic bacteria. The membranes were cut into small pieces, and bacterial DNA was extracted using a commercial kit in accordance with the manufacturer’s protocols. Composite sediment samples were collected from each of the six ponds, resulting in six sediment samples overall. Bacterial DNA was extracted from these sediment samples using the E.Z.N.A.^®^ Soil DNA Kit (Omega Bio-tek, Norcross, GA, United States). 1.0% agarose gel electrophoresis was used to detect DNA integrity, and a NanoDrop2000 UV-Vis spectrophotometer (Thermo Scientific, United States) was used to detect DNA concentration. For Illumina platform sequencing, the V3-V4 region of the 16SrRNA gene was amplified using forward primer 338F (ACTCCTACGGGAGGCAGCAG) and reverse primer 806R (GGACTACHVGGGTWTCTAAT). PCR amplification products were assessed via 2% agarose gel electrophoresis. PCR products were next purified from the gel using the AxyPrep DNA Gel Extraction Kit (AxyPrep Biosciences, Union City, CA, United States) and quantified using a Quantus™ Fluorometer (Promega, United States). Purified PCR products were pooled in equimolar amounts and sent for sequencing. Sequencing was performed on the Illumina MiSeq PE300 platform (Illumina, San Diego, United States) according to the standards of Majorbio bio-pharma Technology Co., Ltd. (Shanghai, China) and the data were analyzed on the Majorbio Cloud platform.^1^ Original FASTQ was quality filtered using FastP and merged with FLASH (Magoč and Salzberg, 2011). Sequences with > 97% similarity were classified into an operational taxonomic unit (OTU) using Uparse (Version 11.0.667) software and the USEARCH11-uparse algorithm (Ma et al., 2023). Species were categorized using the silva138/16s_bacteria database (Release 138)^2^

### 2.5 Co-occurrence network construction

The co-occurrence patterns of sediment and water column bacterial communities under LP and MP treatments were inferred via the SPIEC-EASI algorithm, implemented using the SpiecEasi package in R software (v4.2.5). OTUs detected in half or more than of total samples with relative abundance > 0.01% were kept for network construction (Zhou et al., 2020). To identify interactions among bacterial communities, SparCC results were filtered based on stringent criteria, considering stronger (*R* > 0.75) and significant (*p* < 0.01) coefficients. Network topological parameters, including modularity, average clustering coefficient, average path length, and average degree were computed using the “igraph” R package. The resulting co-occurence networks were visualized using Gephi software. Keystone taxa, pivotal for network structure, were identified base on their within-module connectivity (Zi) and among-module connectivity (Pi). Specifically, nodes were categorized as peripherals (Zi < 2.5; Pi < 0.6), connector(Zi < 2.5; Pi > 0.6), module-hubs (Zi > 2.5; Pi < 0.6), and network hubs (Zi > 2.5; Pi > 0.6) (Olesen et al., 2007). Connectors, module hubs, and network hubs were considered potential keystone taxa due to their influential roles in network topology (Banerjee et al., 2016). The stability of the network was assessed through natural connectivity, which quantifies the rate of robustness reduction upon node removal (Peng and Wu, 2016).

### 2.6 Statistical analysis

Linear mixed-effects models (LMMs) with random intercepts for pond identity were fitted via lmerTest to partition variance and calculate intraclass correlation coefficients (ICC) for repeated sampling data. The null intercept-only model Response∼1+(1| pond) was applied for ICC computation. OLS linear regression was performed and visualized in ggplot2 to show monthly dynamic trends of sediment TN, TC, TP and OM in lotus-fish co-culture (LM) and monoculture (MM) groups, annotated with fitting parameters and 95% regression confidence intervals. Bacterial abundance was counted using Qiime (Version 1.97). Bacterial beta diversity was assessed using Principal Component Analysis (PCA) based on Euclidean distance. Abundance was standardized using the Z-score, and group differences were tested using ANOSIM. Detrended Correspondence Analysis (DCA) was performed using the OTU table at 97% similarity, revealing a substrate bacterial community gradient length of 0.85. Pairwise Bray-Curtis dissimilarity distances were calculated at the OTU level to quantify bacterial community heterogeneity. PERMANOVA with 999 permutations was applied via vegen::adonis2 to partition bacterial community variance constrained by C, N, OM, P, month and nested pond factor based on Bray-Curtis distance. *R*^2^ values represented the explained proportion of community dissimilarity for each variable, and significance was determined at *p* < 0.05. Therefore, redundancy analysis (RDA) was employed instead of canonical correspondence analysis (CCA) to examine the relationship between the sediments bacterial community and sediments organic matter components. Biomarkers in LP and MP were identified by LEfSe analysis using LDA > 3.5 as a threshold (Li et al., 2023). FAPROTAX (Version 1.2.1) were used to prediction of bacterial function. Spearman correlation analysis was used to examine the correlation coefficients between bacterial communities and sediments organic matter in LP and MP.

## 3 Results

### 3.1 Organic matter components of sediments

**FIGURE 1:**
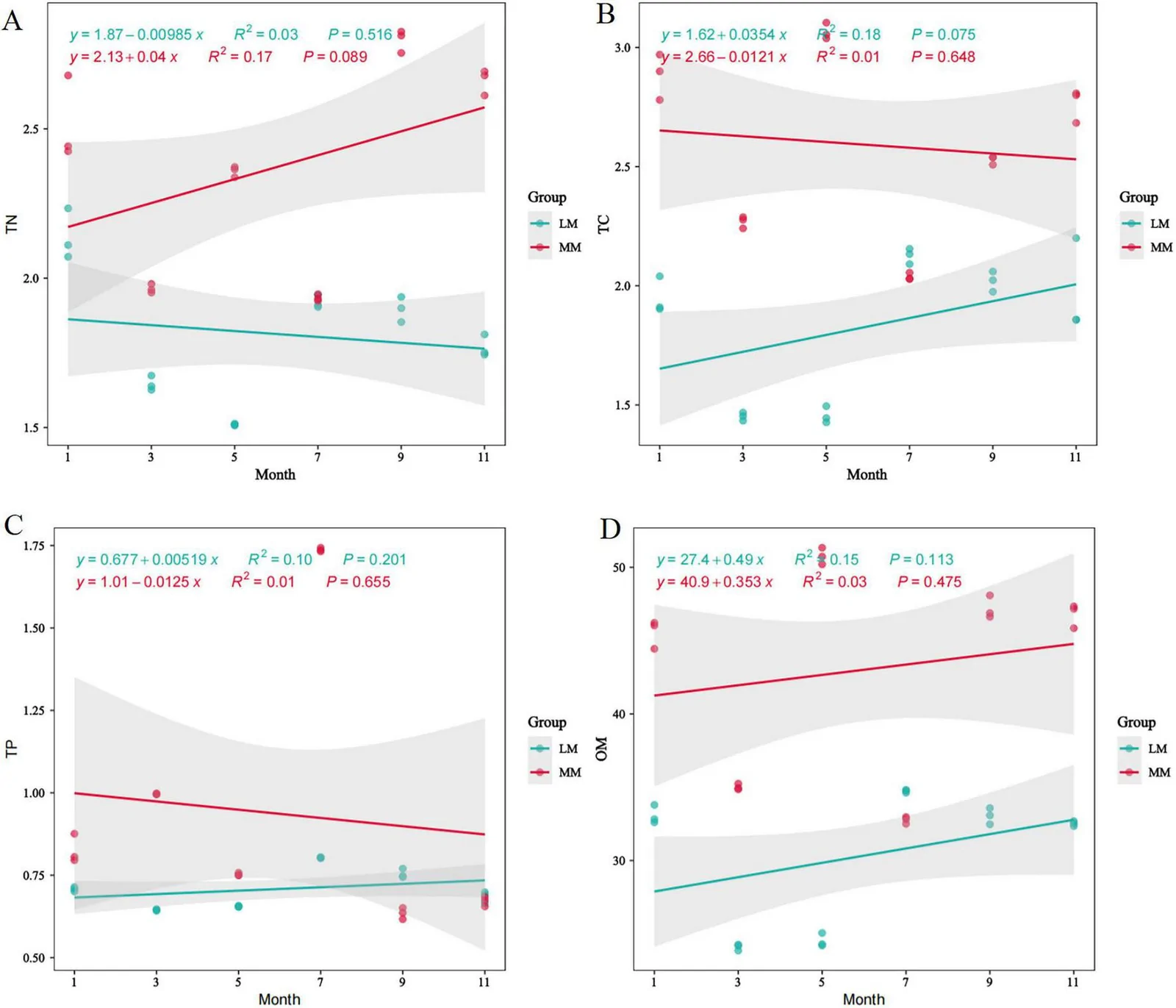
**(A)** Total nitrogen (TN). **(B)** Total carbon (TC). **(C)** Total phosphorus (TP). **(D)** Organic matter (OM). Lines represent OLS linear regression fits for each group, with annotated regression formula, coefficient of determination (R^2^) and *P*-value. Gray shaded areas indicate the 95% confidence intervals of regression models. Linear mixed-effects models (LMMs) with pond as a random intercept were additionally used to calculate intraclass correlation coefficient (ICC) and account for repeated-measure pseudoreplication across sampling months.

We used univariate linear regression to analyze monthly dynamics of sediment TN, TC, TP, and OM in LP and MP ponds. No significant linear temporal trends were detected for all nutrients in MP (*p* > 0.05), which fluctuated irregularly at relatively high concentrations throughout the culture period. In LP, TN slightly accumulated in July but showed a marginally significant decreasing trend over time. TP and OM declined temporarily from January to March and increased slowly in the whole year, with *p*-values of 0.075 and 0.113, respectively (Figure 1).

### 3.2 Identification of algae and determination of chlorophyll *a* concentration

**FIGURE 2:**
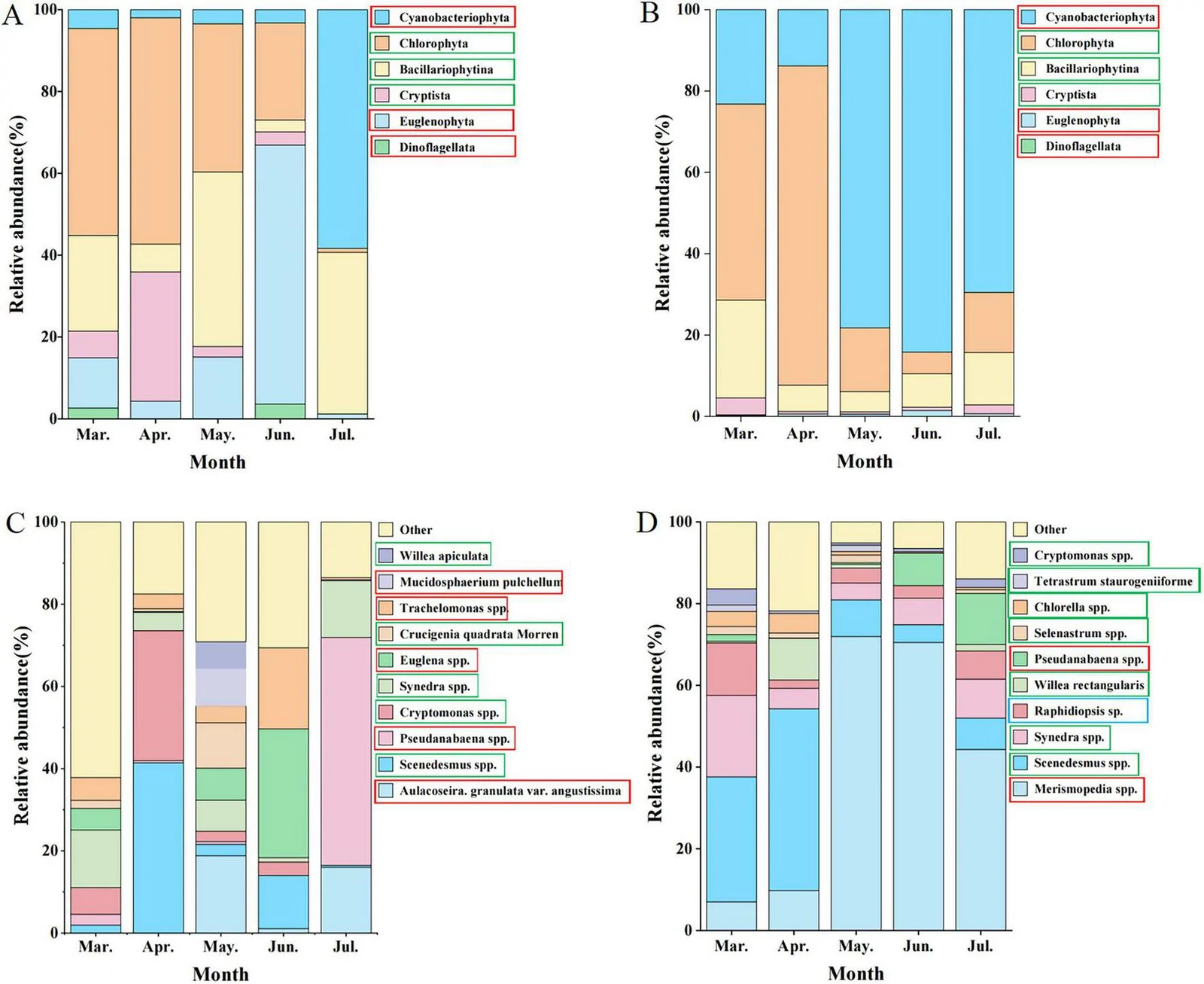
Algae community composition. **(A,B)** The algae community composition at the phylum level for LP and MP, respectively. **(C,D)** The phytoplankton community composition at the species level for LP and MP, respectively. Algae marked by red boxes in the figure represent harmful taxa, those in green boxes are beneficial algae, and blue boxes indicate neutral-status algae.

Six algae phylum (*Cyanobacteriophyta*, *Chlorophyta*, *Bacillariophytina*, *Cryptista*, *Euglenophyta*, and *Dinoflagellata*) were identified in both LP and MP. The composition of the algae community varied monthly in LP, while *Cyanophyta* and *Chlorophyta* dominated in MP (Figures 2A,B). At the species level, the two pond groups also showed differences. A total of 67 algae species were identified in LP, with dominant species including *Aulacoseira. granulata var. angustissima (O. Müll.) Simonsen*, *Scenedesmus* spp., *Pseudanabaena* spp., and *Cryptomonas* spp.*, among others*. In MP, 62 algae species were identified, with dominant genera including *Merismopedia* spp., *Scendesmus* spp., *Synedra* spp., and *Raphidiopsis* sp., among others (Figures 2C,D).

The concentration of chlorophyll *a* in LP peaked in July, fluctuating between 8 and 37 mg/m^3^, while in MP, it peaked in May and fluctuated between 20 and 126 mg/m^3^, indicating a higher algae abundance in MP (Supplementary Figure 2).

### 3.3 Diversity and composition of bacterial community

**FIGURE 3:**
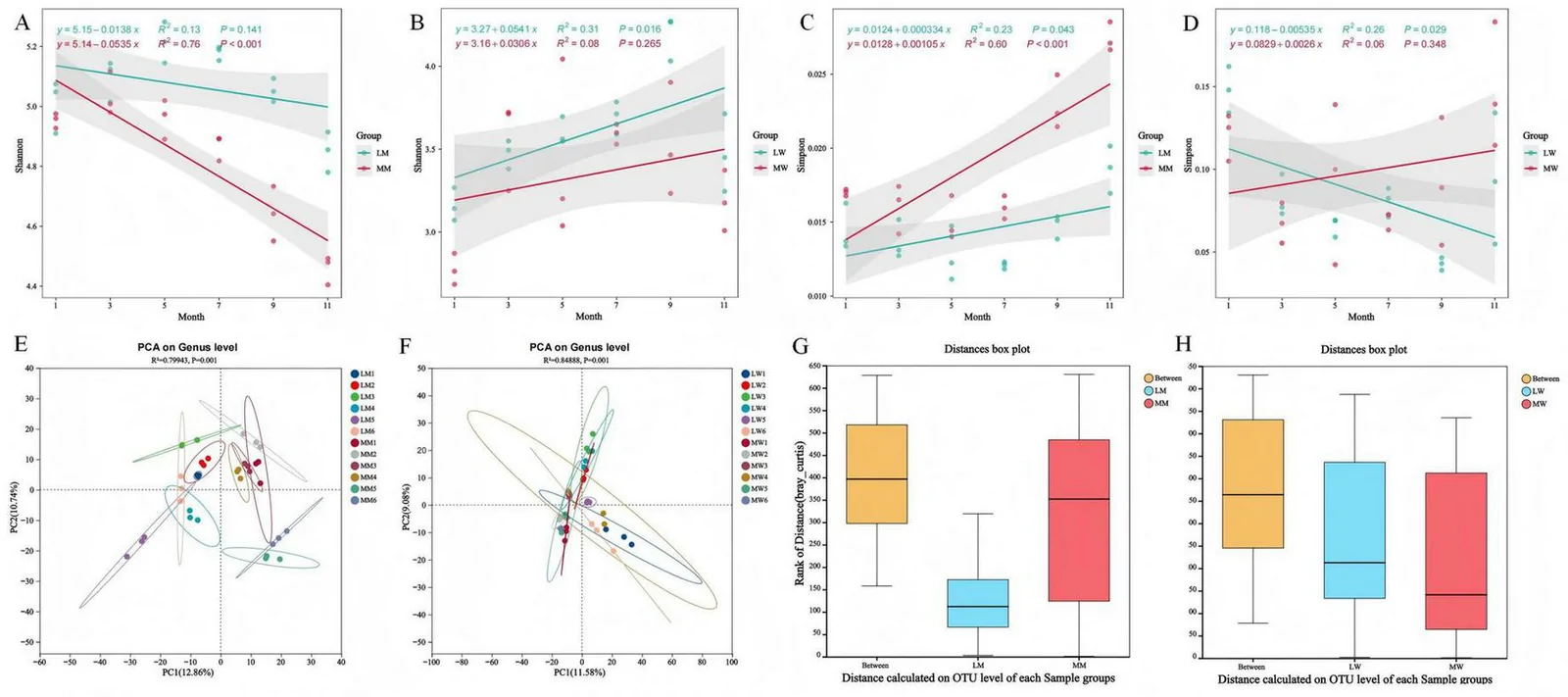
**(A–D)** OLS linear regression fits of Shannon and Simpson indices across sampling months, with regression parameters and 95% confidence intervals displayed. LMM-based ICC calculation with pond random intercept was adopted uniformly for diversity indices. **(E,F)** Genus-level PCA coupled with ANOSIM test for community separation. **(G,H)** Ranked Bray-Curtis distance boxplots comparing inter- and intra-group bacterial community heterogeneity at the OTU level.

Linear regression was used to assess monthly dynamics of Shannon and Simpson Alpha-diversity indices under different aquaculture models, with obvious divergent temporal succession observed between two systems (Figure 3). In LM, Shannon slightly decreased non-significantly (*R*^2^ = 0.13, *p* = 0.1413), while Simpson rose significantly (*R*^2^ = 0.23, *p* = 0.043) (Figures 3A,C). MM showed an extremely significant drop in Shannon (*R*^2^ = 0.76, *p* < 0.001) and extreme increase in Simpson (*R*^2^ = 0.60, *p* < 0.001) (Figures 3A,C). LW had a significant upward Shannon trend (*R*^2^ = 0.31, *p* = 0.016) and significant downward Simpson trend (*R*^2^ = 0.26, *p* = 0.029) (Figures 3B,D). No significant trends were detected for both indices in MW(Figures 3B,D).

PCA was performed on z-score normalized bacterial abundance data from sediment and water samples across 6 months, with 95% confidence ellipses illustrating sample distribution (Figures 3E,F). Sediment bacterial communities exhibited highly significant intergroup separation (*R*^2^ = 79.94%, *p* = 0.001) with clear monthly clustering (Figure 3E), while water microbiota also showed extremely significant grouping divergence (*R*^2^ = 84.89%, *p* = 0.001) and obvious seasonal succession(Figure 3F). Water communities in lotus-fish co-culture ponds (LP) followed regular seasonal gradients, with highly similar assemblages in winter and remarkable separation along PC1 in summer. In monoculture ponds (MP), dramatic community shifts only occurred in early spring (January–March), and the structure remained stable in other months. ANOSIM based on OTU-level Bray-Curtis distances confirmed significant community dissimilarity between LM vs. MM and LW vs. MW (*R* = 0.57, *P* < 0.05) (Figures 3G,H). PERMANOVA further identified aquaculture mode as the primary driver of β-diversity variation (Supplementary Tables 1, 2). For sediment (LM/MM), different pond explained 22.21% of total variance (*F* = 9.71, *P* = 0.001); for water (LW/MW), it accounted for 19.53% variation (*F* = 8.25). Both values far exceeded the explanatory power of sampling month (5.70 and 16.58%, respectively). Sediment TC, TN, TP and OM all imposed significant constraints on community composition (*P* < 0.01), among which TN showed the highest independent explanation (*R*^2^ = 0.140), implying that Ponds mediated microbial differentiation indirectly by regulating nutrient levels. Collectively, stable structural discrepancies existed between co-culture and monoculture microbiota, and aquaculture mode outweighed seasonal dynamics in shaping community assembly.

**FIGURE 4:**
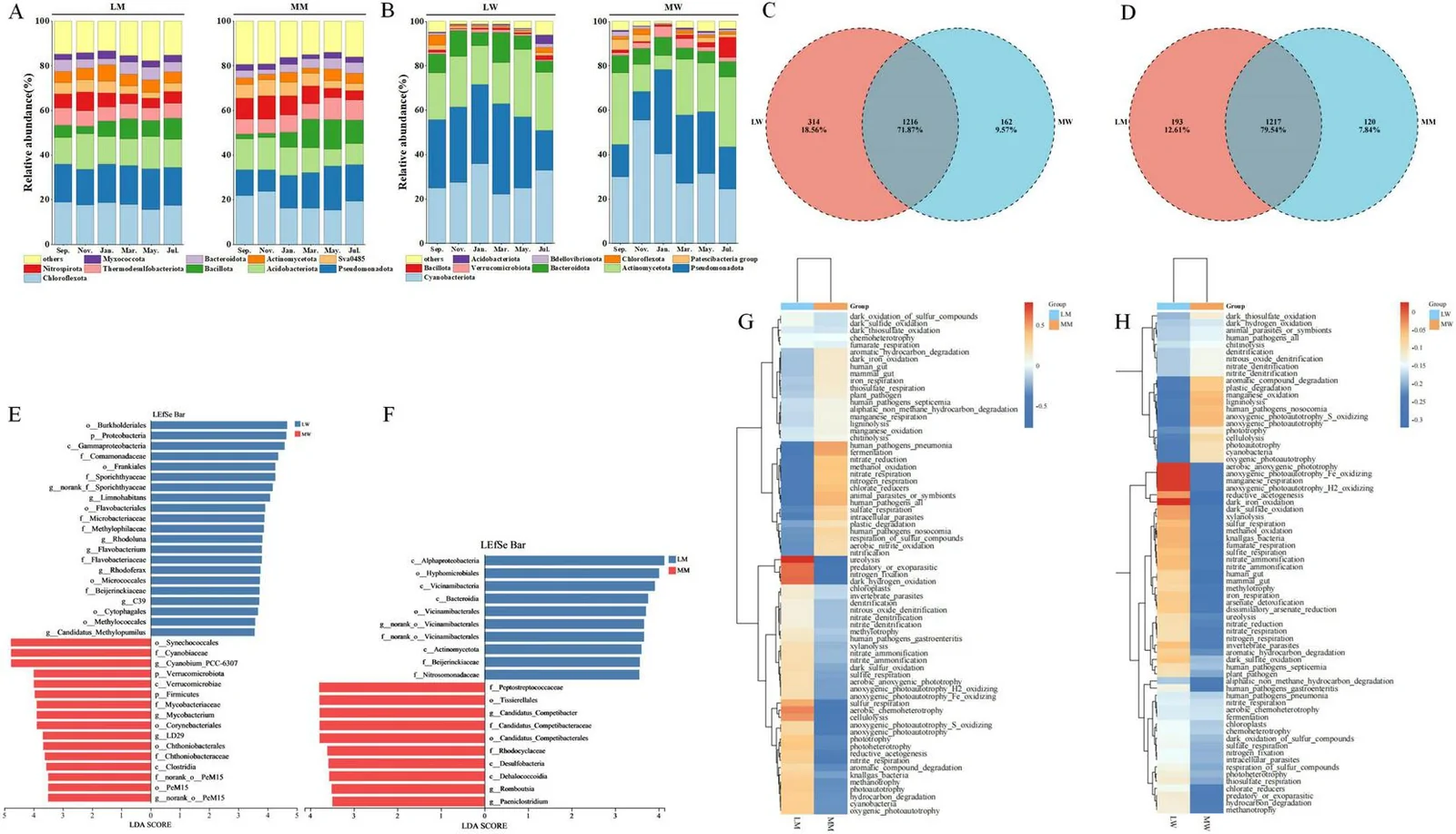
**(A,B)** Stacked bar charts showing the relative abundance of dominant bacterial phyla at the phylum level in LM-MM and LW-MW paired groups, respectively. Each color represents one bacterial phylum. **(C)** Shared genu between LW and MW. **(D)** Shared genu between LM and MM under co-culture and monoculture modes. **(E,F)** Linear discriminant analysis effect size (LEfSe) bar plots showing significantly differential biomarker taxa with an LDA score ≥ 3.5. Panel E represents the comparison between LM and MM, and Panel F represents the comparison between LW and MW. Blue bars denote taxa enriched in the lotus-fish co-culture group, whereas red bars indicate taxa significantly enriched in the monoculture group. **(G,H)** Heatmaps of normalized predicted functional pathway abundance derived from the whole bacterial community (all detected genera) for LM-MM and LW-MW comparisons. The color gradient corresponds to standardized functional relative abundance.

The dominant phylum in the sediments of both pond groups were *Chloroflexota*, *Pseudomonadota* and *Acidobacteriota*; and the water dominantd with *Cyanobacteriota*, *Pseudomonadota*, *Actinomycetota*, and *Bacteroidota* (Figures 4A,B). The dominant genus in LP sediments were *g__norank_c__Subgroup_18*, *g__norank_c__Thermodesulfovibrionia*, and etc., whereas the dominant genus in the water column including *g__CL500-29_marine_group*, *g__Cyanobium_PCC-6307*, *g__Flavobacterium*, and etc. (Supplementary Figures 3A,B). The dominant geuns in MP sediments were *g__Clostridium_sensu_stricto_1*, *g__norank_c__Thermodesulfovibrionia*, and etc., whereas the dominant genus in water column including *g__Acinetobacter*, *g__Mycobacterium*, and etc. (Supplementary Figures 3C,D).

### 3.4 Biomarkers and bacterial function analysis between the LP and MP

The LP sediments contains 1,410 geuns, while the MP sediments has 1,337 genus. The shared bacterial community between the two groups comprises 1,217 genus. The LP water contains 1,530 genus, whereas the MP water has 1,378 genus. The shared bacterial community between the two groups includes 1,216 genus. The number of shared bacteria between the two pond groups’ sediments and water remains similar without significant changes, though the LP sediments and water contain more unique bacteria groups than the MP (Figures 4C,D).

LEfSe analysis showed that there were 35 biomarkers with significant differences from class to genus in the sediment samples of the two pond groups. There were 21 biomarkers with significant difference in LP, including *c*_*Gammaproteobacteria*, *o*_*Frankiales*, *o*_*Burkholderiales*, *g*_*Candidatus_Methylopumilus*, *g*_*Rhodoluna*, and etc.. There are 14 biomarkers with significant difference in MP, among which the dominant bacteria are *g*_*Cyanobium_PCC-6307*, *g*_*Mycobacterium*, *g*_*LD29*, etc. (Figure 4E).

Similarly, a total of 20 biomarkers with significant differences from class to genus were detected in the water samples of the two pond groups. There were 11 biomarkers with significant differences in LP, among which they were: *c*_*Alphaproteobacteria*, *o__Hyphomicrobiales*, *c*_*Vicinamibacterales*, etc.; There were 9 biomarkers with significant differences in MP sediments, and they were *o__Candidatus_Competibacterales*, *g_Romboutsia*, *c_Desulfobacteria*, *g_Candidatus_Competibacter*, etc. (Figure 4F).

In LP, Methylotrophy and methanotrophy were enriched. Its nitrogen cycle covered nitrogen fixation, multi-step denitrification, ammonification, nitrite respiration and ureolysis, with denitrification, one-carbon metabolism and versatile phototrophy as the functional core. By contrast, MP sediment harbored a divergent functional profile featured by metal redox transformation, sulfur metabolism and nitrification-dominated nitrogen metabolism. Multiple dark sulfur oxidation and sulfur respiration pathways endowed strong sulfur conversion capacity. Nitrogen metabolism was dominated by nitrification. Furthermore, MP was enriched in extensive host-related functions, such as mammalian gut symbiosis, plant and human pathogens, as well as animal parasites or symbionts (Figure 4G). For carbon metabolism, LP water was enriched in carbon degradation and one-carbon metabolic pathways including xylanolysis, hydrocarbon degradation and methanotrophy. In terms of light energy metabolism, multiple anoxygenic photoautotrophic systems using Fe and H_2_ as electron donors were detected. It also possessed a complete respiratory network containing metal respiration, sulfur respiration, nitrogen respiration and fumarate respiration, conferring strong capacities for energy me olism and material transformation. In contrast, MP water harbored a relatively simple functional profile dominated by phototrophy, organic matter/polysaccharide degradation, and characteristic manganese oxidation. Its photoautotrophic processes mainly relied on sulfur compounds as substrates (Figure 4H).

### 3.5 Co-occurrence network patterns of bacterial communities

**FIGURE 5:**
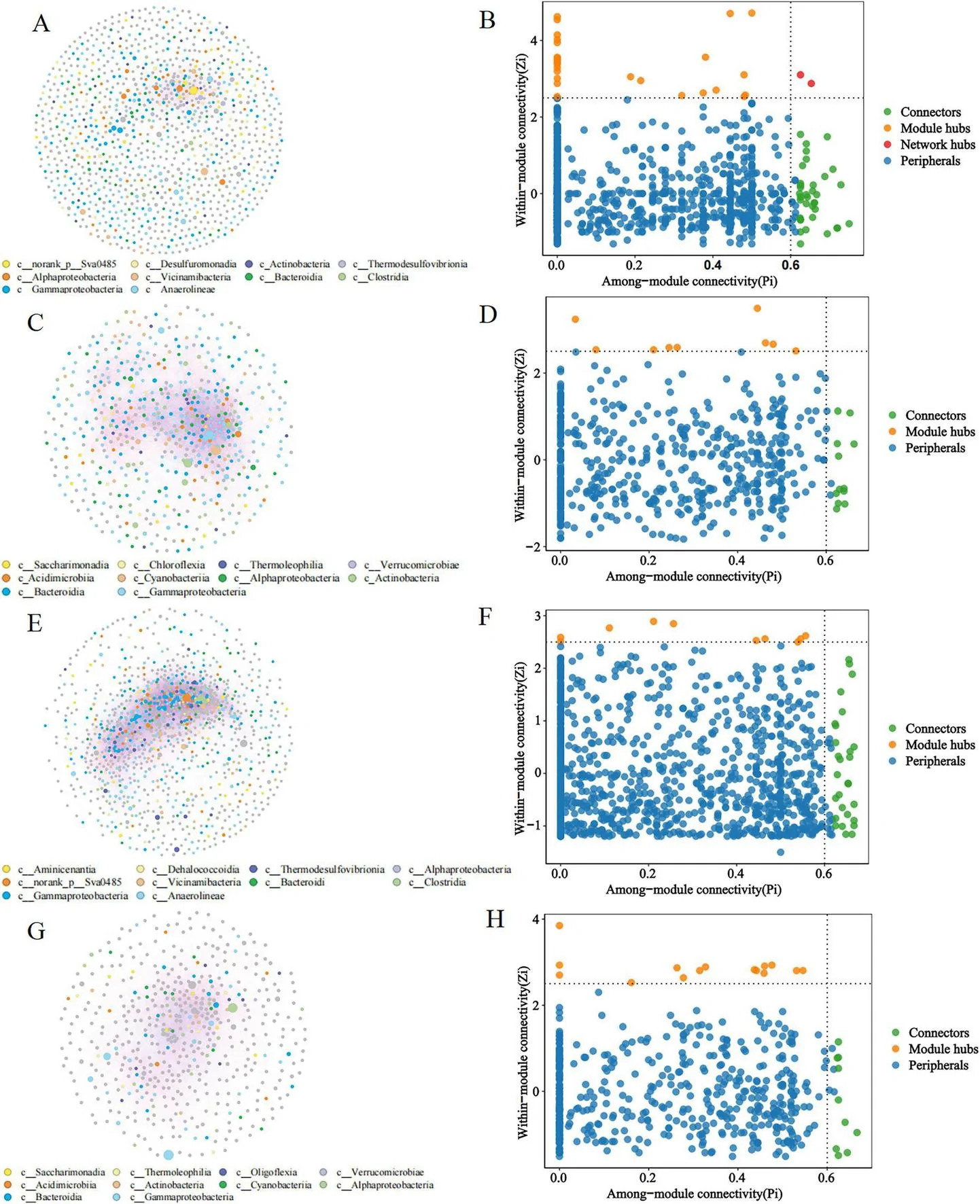
Co-occurrence network of bacterial communities in the sediment and water of two aquaculture ponds. Networks were colored by the top 10 bacterial classes in relative abundance. Node size is the total abundance of each OTU to distinguish from bacterial nodes. **(A,C,E,G)** Genera-level bacterial co-occurrence networks colored by taxonomic groups. **(B,D,F,H)** Zi-Pi plots partitioning nodes into four topological categories (Module hubs, Network hubs, Connectors, Peripherals) using standard thresholds Zi = 2.5 and Pi = 0.62.

Compared to LM, the MM, LW, and MW networks exhibited higher edge counts. Although LM possessed more nodes, its network structure was relatively sparse (Figures 5A,B and Supplementary Table 3). Meanwhile, while MM, LW, and MW networks demonstrate lower modularity (Figures 5C–H and Supplementary Table 3). Additionally, LW, MW, and MM networks exhibit higher average clustering coefficients and shorter average path lengths (Supplementary Table 3). In contrast, LM networks demonstrate lower average clustering coefficients and longer path lengths Meanwhile, the significantly higher average degree of MM, LW, and MW (Supplementary Table 3). At the node-topology role level, the LM network identified 36 connectors, 26 module hubs, and 2 network hubs, with the highest number of keystone taxa (Supplementary Table 4).

**FIGURE 6:**
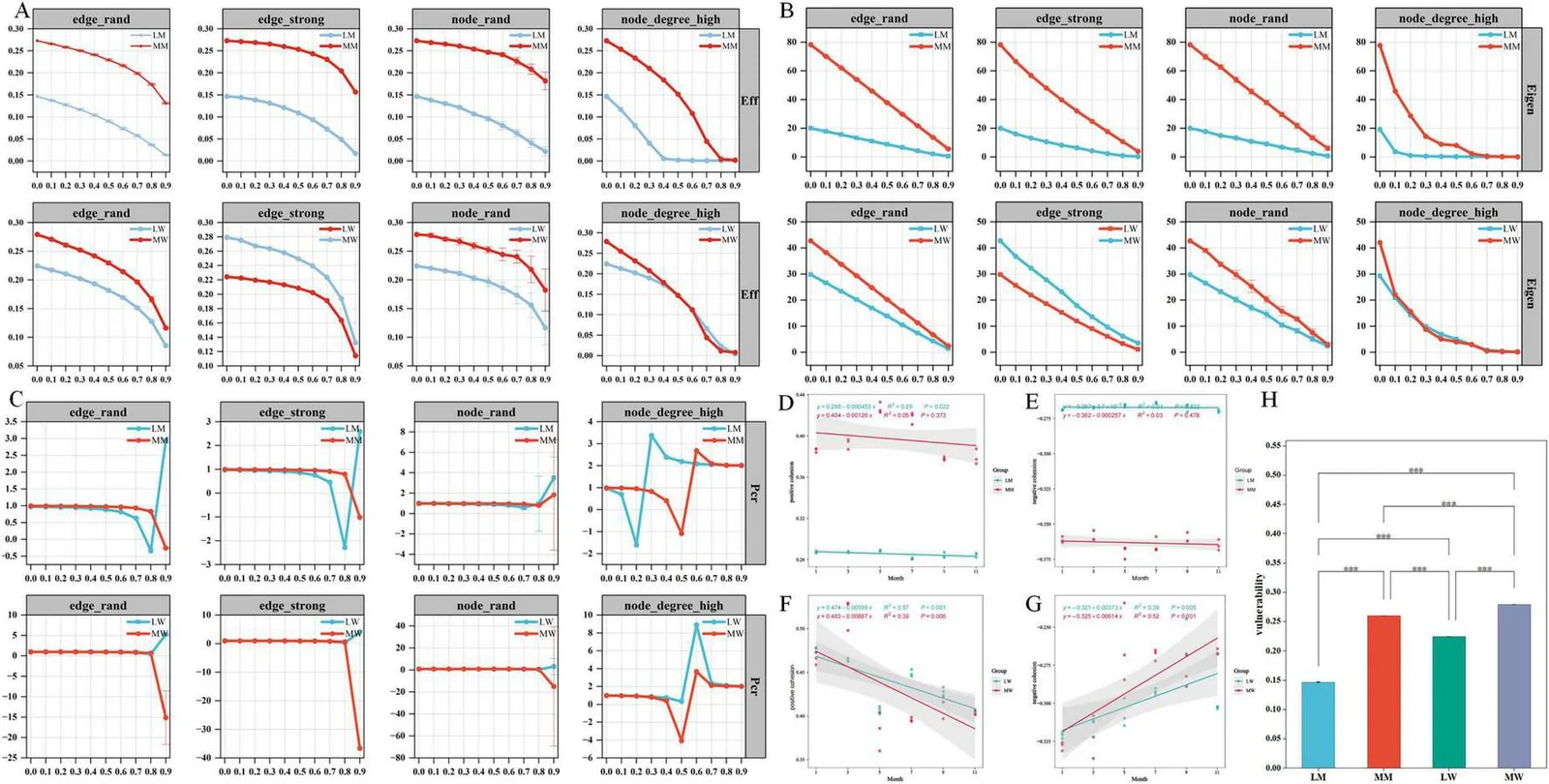
(A–C) Network robustness evaluation under random edge removal, strong edge removal, random node removal and high-degree node targeted removal for LM-MM and LW-MW comparisons, respectively. The vertical axis represents natural connectivity of the network, and the horizontal axis denotes the proportion of removed edges/nodes. **(D–G)** Linear regression fitting of monthly positive cohesion and negative cohesion indices in paired treatments, with regression equations, R2 and *p*-values annotated on the fitting lines. **(H)** Bar chart showing intergroup differences in vulnerability network cohesion index among four groups, with significant pairwise differences labeled.

Under random perturbation and targeted attack on core network structures, the decline rates of network efficiency and eigenvalue decay in MM and MW networks were significantly slower than those in LM and LW networks. Especially under directional removal of high-degree hub nodes, the connectivity of LM and LW collapsed rapidly, whereas MM and MW maintained favorable network connectivity. The structural preservation rate (Pcr) further verified that intense disturbance caused massive topological destruction of LM and LW networks (Figures 6A–C).

For sediment bacterial communities, the strength of positive cohesion among taxa in the MM decreased significantly with the progression of the aquaculture cycle; the LM sustained a low level of positive cohesion throughout the whole year. In contrast, positive cohesion of water bacterial communities (LW and MW) declined sharply over months, accompanied by continuous and significant elevation of negative cohesion. Notably, MW exhibited a faster rising rate and higher explanatory power for negative cohesion than the LW (MW: *R*^2^ = 0.52, *P* < 0.001; LW: *R*^2^ = 0.39, *p* = 0.005) (Figures 6D–G).

### 3.6 Correlation analysis between the sediments bacteria and sediments organic matter components

**FIGURE 7:**
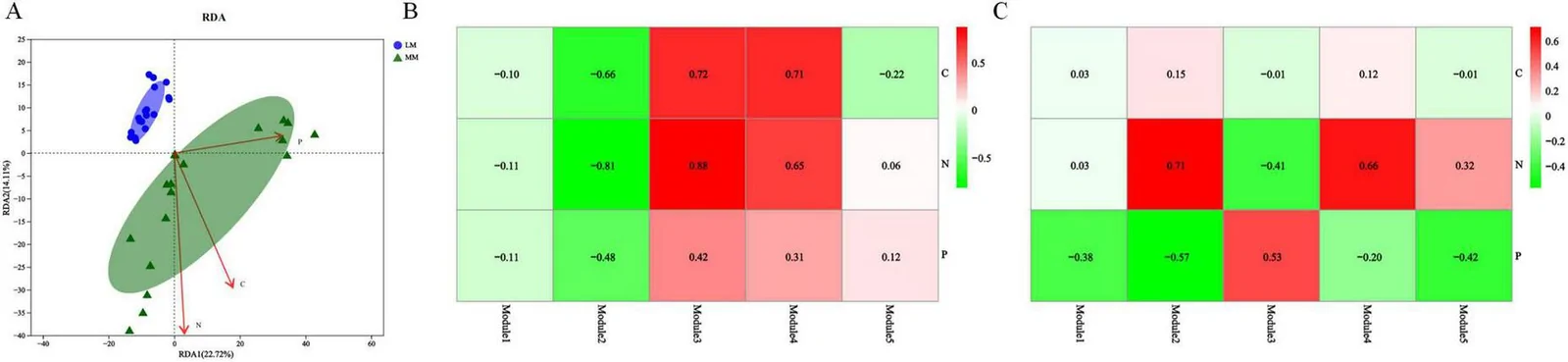
**(A)** RDA ordination plot showing the differentiation of bacterial community structure constrained by sediment total carbon (TC), total nitrogen (TN) and total phosphorus (TP). Blue dots represent LM samples, green triangles represent MM samples. Red arrows indicate the environmental vectors of C, N, P. The percentage on each axis represents the explained variation of community dissimilarity. **(B,C)** Pearson correlation heatmaps between three sediment nutrient variables (C, N, P) and reads abundance of five network modules for LM and MM groups, respectively. Red color indicates positive correlation, green color indicates negative correlation, and the numerical value in each cell is the correlation coefficient.

Prior to redundancy analysis (RDA), we eliminated highly autocorrelated environmental variables via variance inflation factor (VIF) analysis to retain representative factors. The VIF values of TC, TN, TP and OM were calculated as 21.47, 7.56, 1.13, and 37.71, respectively. Strong collinearity existed between TC and OM, so OM was removed from the dataset. A second round of VIF assessment on TC, TN and TP yielded values of 3.42, 3.47, and 1.04, all below the threshold of 10. Consequently, TC, TN and TP were selected for subsequent RDA. The RDA results showed that the sediments organic matter components (TC, TN, TP) were positively correlated with the MP sediments bacteria, and negatively correlated with the LP sediments bacteria. These elements in the sediments explain a significant portion (36.83%) of the variation in bacterial community structure, with RDA1 accounting for 22.72% and RDA2 for 14.11% (Figure 7A). RDA results indicated that TC and TN were strong drivers shaping the bacterial community in MM, while TP showed negative correlations with partial microbial taxa. We further performed WGCNA analysis linking modules partitioned from the bacterial co-occurrence network to environmental factors, and displayed the top five core modules with the largest node sizes (Figures 7B,C). In LP ponds, Module 3 and Module 4 exhibited the strongest responses to carbon and nitrogen loading, consistent with the dominant functions of nitrogen removal and organic matter degradation in LP sediments. Module 1 showed negligible correlations with TC, TN and TP, implying its assembly was governed by other environmental variables (Figure 7B). For MP ponds, TN was the primary nutrient driver; Module 2, Module 4 and Module 5 were positively associated with TN, while TP exerted largely negative impacts on most modules, and TC had weak regulatory effects on bacterial modules (Figure 7C). Count statistics of module-nutrient correlations revealed that TC, TN and TP drove extensive differentiation of bacterial modules in LP, with TP exerting predominantly positive effects. In MP, TC showed weak regulatory capacity, TN acted as the major positive driver, and TP suppressed most modules, resulting in distinct microbial phosphorus responses between the two culture systems.

### 3.7 Comparison of fish growth between two pond groups

At 1 year, LP group exhibited greater body weight (707.37 ± 37.39 g) and length (31.28 ± 0.656 cm) than MP group (640.10 ± 35.32 g, 29.04 ± 0.81 cm) (*p* < 0.05). CF were significantly lower in LP (*p* < 0.001), with no difference in WGR and SGR (*p* > 0.05) (Table 1).

**TABLE 1:** Two-group comparison of growth performance of 1-year-old fish in two types of aquaculture ponds.

Group growth	LP		MP		*P*
	Mean	SD	Mean	SD	
FBW	707.37	37.39	640.10	35.32	< 0.0001
FBL	31.28	0.66	29.04	0.81	< 0.0001
WGR	8.81	1.04	8.02	1.97	0.23494
SGR	0.0063	3.07E-4	0.0061	5.89E-4	0.1654
CF	0.0231	6.22E-4	0.0261	0.00107	< 0.0001

Values are presented as mean ± standard error (*n* = 12). P denotes the *p*-value, and a value of *p* < 0.05 indicates a significant difference. FBW (g) was final body weight. FBL (cm) was average final body length. WGR (weight gain rate, %) = (FBW - IBW)/IBW × 100 %. SGR (specific growth rate, %/day) = [ln (FBW) - In (IBW)]/days × 100 %. Condition factor (CF) was used to evaluate the body condition and plumpness of fish. It was calculated as: k = W/L^3^× 100%. where W is the body weight (g) and L is body length (m).

## 4 Discussion

Horizontal comparison between groups revealed that the annual background nutrient concentrations in the LP group were generally lower than those in the fish monoculture group (Figure 1). Although phosphorus and organic matter gradually accumulated during the culture period, this Lotu-fish co-culture mode could effectively reduce the total nitrogen load of the system. Similar seasonal fluctuations of nutrients were also reported in the polyculture system of yellow catfish and lotus by Yang et al. (2022a). Combined with lotus phenology, the variation could be reasonably explained. During the rapid seedling growth stage (March–June), vigorously growing lotus roots assimilated sediment ammonium nitrogen, nitrite nitrogen and soluble phosphorus, which were stored in rhizomes and leaves, jointly reducing sediment TN, TP and OM. In July when lotus bloomed and fruited, abundant senescent litter settled into sediment, increasing exogenous organic matter and causing temporary nutrient accumulation. From August to November, tuber harvesting exported nitrogen, phosphorus and organic compounds out of the pond ecosystem. Exudation from lotus rhizomes enhanced carbon utilization efficiency of sediment microorganisms, which was indirectly evidenced by the September AWCD (Supplementary Figure 1) results and consistent with Zhang et al.’s research (Zhang et al., 2013). In November, lotus went into overwintering dormancy with reduced nutrient absorption capacity. TN, TP and OM declined marginally yet stayed above the levels of the early seedling stage, resulting in minor net nutrient accumulation throughout the farming cycle. Collectively, these results indicated that the lotus-fish ecological polyculture possessed considerable potential for regulating nutrients in aquaculture water and sediments.

In MP, the phylum level was dominated by *Chlorophyta* and *Cyanobacteriophyta*, while the species level was dominated by *Merismopedia* spp. and *Scenedesmus* spp., one of the former species for water quality pollution (Radwan et al., 2018). The composition of dominant algae in the LP varied from month to month at the phylum, suggesting that the ecological niche occupied by different algae in the LP changes significantly as with time (Figures 2A,D). Also, the concentration of chlorophyll *a* in LP was lower than that in MP throughout the study, peaking in July (Supplementary Figure 2), consistent with findings by Yuan et al. in their study of a lotus-yellow catfish co-culture system (Yuan et al., 2019). Lotus plants affect water temperature, light penetration and organic matter content. Compared with bare open water, lotus-covered ponds impose stronger environmental disturbances and consequently alter aquatic habitat conditions (Liu and Qiu, 2007). Phytoplankton communities in LP ponds showed obvious seasonal succession, likely driven by environmental disturbances. *Chlorophyta* with broad temperature adaptability dominated March–June (Maberly et al., 2022). *Bacillariophyta* and *Cryptophyta* prevailed in spring; low temperature and water turbulence supplemented nutrients and boosted diatom abundance in March and May (Lu et al., 2017). Flagellated Euglenophyta favored organic-rich water and became dominant in June(Han et al., 2022). High temperature, strong light and high nutrients triggered cyanobacterial blooms in July, establishing Cyanobacteria as the dominant group. *Chlorophyta* including *Chlorella* spp., *Scenedesmus* spp., and *Crucigenia quadrata* form a favorable algal community in aquaculture water (Liu et al., 2023; Zhang et al., 2026). They increase dissolved oxygen, promote nutrient transformation, reduce organic pollutants, and supply natural food containing essential n-6 polyunsaturated fatty acids for aquatic organisms (Kim et al., 2021). Several non-toxic *Bacillariophyta* (*Willea apiculata*, *Willea rectangularis*, *Synedra* spp.) are rich in EPA and DHA and serve as high-quality natural food that sustains fish growth (Zhang et al., 2019). *Aulacoseira granulata var. angustissima* acts as a bio-indicator of eutrophic-polluted water environments, whose population prosperity responds sensitively to water-temperature variation and excessive nutrients. As another valuable beneficial alga, *Cryptomonas* spp. owns the highest n-3/n-6 fatty-acid ratio among freshwater phytoplankton and bears no toxin-producing risk (Napiórkowska-Krzebietke, 2017). *Euglena* sp. and *Trachelomonas* sp. are typical conditional-neutral indicator algae (Sultana et al., 2024). They frequently develop seasonal surface blooms under organic-rich eutrophic circumstances without stable toxicity. By comparison, cyanobacteria such as *Pseudanabaena* spp. and *Merismopedia* spp. are classified as harmful algae (Zhu et al., 2015). They may yield anatoxins and cyanotoxins, trigger dense algal-bloom outbreaks and damage aquatic-organism health and ecosystem stability. Among these algae, *Chlorophyta* and *Bacillariophyta* accumulate abundantly in the LP group. Serving as fundamental food algae, they supply grass carp with abundant amino acids, polysaccharides and fatty acids and thereby facilitate fish growth.

The Shannon index is commonly used to assess bacterial diversity. Higher bacterial Alpha-diversity has been linked to increased growth rates and improved health in fish (Almeida et al., 2021). Shannon and Simpson indices are widely used to evaluate bacterial alpha diversity, and higher Alpha-diversity correlates with better fish growth and health status (Wang et al., 2020). The LP had higher Shannon indices and lower Simpson indices in both sediment and water bacterial communities. The sediment bacterial Shannon diversity index declined after July under both aquaculture modes, with different potential driving mechanisms (Figures 3A–D). In the LP group, lotus harvesting removed considerable TN, TC, TP and organic matter. Reduced sediment nutrients may aggravate bacterial resource competition and reduce community diversity. For the MP group, TC, TN and OM accumulated greatly in the later stage while TP was markedly depleted, implying phosphorus might be a critical limiting factor; fierce competition for phosphorus could simplify bacterial community structure. The diversity decreased far more gently in LP than MP, possibly owing to the regulatory role of lotus. Lotus litter and root exudates provide diverse carbon substrates that support the coexistence of various bacteria, superior to the single organic input from feed residues and fish feces in MP (Ström et al., 2012). Meanwhile, root radial oxygen loss improves sediment habitat heterogeneity, relieves environmental filtering and slows the loss of bacterial diversity.

Beta-diversity analysis revealed that aquaculture model was the primary driver shaping the bacterial community structure in both sediments and water columns (Figures 3E–H). In the sediment of the LP pond, *g*_*Candidatus Methylopumilus* mediates nitrate assimilation, methanol oxidation and sulfate transport (Layoun et al., 2024; Salcher et al., 2025); *g*_*Rhodoferax* indirectly regulates sediment methane emission via iron reduction coupled with denitrification (Beattie et al., 2020); *g*_*Rhodoluna* drives the cycling of organic carbon, nitrogen and reduced sulfur relying on the rhodopsin-based photosynthetic system (Hempel et al., 2021; Peoples Logan et al., 2023); *g*_*Limnohabitans* optimizes the sediment microbial food web through dual heterotrophic metabolism (Kasalický et al., 2018); *f*_*Sporichthyaceae* degrade humus to improve community stability in winter and facilitate nutrient turnover under low-temperature conditions (Liu et al., 2025); *f*_*Flavobacterium* decomposes macromolecular organic matter derived from animals and plants to supply substrates for methanogens, thereby alleviating the accumulation of organic matter in sediment (Ma et al., 2025; Zhao et al., 2025). In the water column of the LP pond, *f*_*Beijerinckiaceae* possess both methanotrophy and biological nitrogen fixation capacity, acting as the pivotal taxa coupling carbon and nitrogen cycles (Kolton et al., 2022). *c*_*Vicinamibacteria* harbor potential phosphorus-solubilizing capacity to mobilize inert phosphorus in sediment (Wu et al., 2021). *f*_*Nitrosomonadaceae* catalyze ammonia oxidation, the rate-limiting step of nitrification, converting highly toxic ammonia nitrogen into nitrate nitrogen and optimizing the nitrogen pool of water bodies (Clark et al., 2021). Sulfur-degrading functional bacteria realize the coupling of sulfur and nitrogen cycles and accelerate the degradation of endogenous pollutants in the pond. For the MP pond, *g*_*Cyanobium_PCC-6307* dominates the sediment in summer. Its massive proliferation under high temperature and strong light tends to exacerbate the risk of eutrophication (Xu et al., 2025). In MP water, the class *c*_*Desulfobacteria* (sulfate-reducing bacteria, SRB) are obligate anaerobic microbes that drive dissimilatory sulfate reduction coupled with nitrate transformation to reduce nitrate accumulation (Dyksma and Pester, 2024). *g*_*Candidatus_Competibacter* (glycogen-accumulating organisms, GAOs) may impair the biological phosphorus removal efficiency of the ecosystem and cause phosphorus retention (Brand Veronica et al., 2019). The class *c*_*Dehalococcoidia* degrade refractory halogenated organic compounds through reductive dechlorination. Functional profiles matched microbial biomarker characteristics (Palmer et al., 2023). The genus *g*_*Mycobacterium* detected in monoculture ponds is an opportunistic pathogen (Delghandi et al., 2020). It causes chronic granulomatous mycobacteriosis in fish under poor sediment and high nutrient loads, poses zoonotic risks, and acts as a bioindicator of degraded benthic health. The LP system exhibited stratified metabolic cooperation: sediment dominated organic decomposition and denitrification-based nitrogen cycling, while the water column conducted carbon mineralization and redox metabolism, jointly maintaining stable C–N–S coupling. In comparison, MP sediment was characterized by recalcitrant organic degradation, nitrification predominance, intensive Fe–Mn and sulfur cycling, as well as abundant symbiotic and pathogenic functions. MP water showed greatly simplified metabolism with sulfur-dependent photoautotrophy, differing from the iron- and hydrogen-based phototrophy in LP water. Variations in sediment organic matter input and plant-mediated nutrient retention drove distinct bacterial functional profiles between the two aquaculture models. The lotus–fish co-culture established stratified sediment-water C–N–P metabolic coupling, enriching diverse functional bacteria capable of efficient nitrogen removal, internal phosphorus activation, and organic matter degradation. In contrast, the mono-fish culture created anaerobic sediment and high-light water conditions that favored cyanobacteria, sulfate-reducing bacteria, and glycogen-accumulating organisms. This system exhibited simplified metabolism, nitrogen and phosphorus retention, and dominant sulfur-driven anaerobic cycling, ultimately differentiating bacterial community assembly and elemental cycling strategies.

Network analysis of bacterial co-occurrences, as measured by abundance correlation of OTUs, can help decipher bacterial association (Figure 5). After filtering by relative abundance, the LP network contained more nodes and edges yet exhibited a sparser topological structure, whereas the MP network had a higher average clustering coefficient and shorter average path length (Supplementary Table 3). In MM, MW, and LW, interactions within taxa may enhance community recovery following disturbances, thereby buffering the impacts of environmental fluctuations (Konopka et al., 2014). We further conducted network robustness analyses and revealed that the MP community underwent slower degradation upon the removal of edges, nodes, strong edges or strong nodes (Figures 6A–C). In contrast, the LP community possessed higher intra-community negative cohesion and a looser network topology, indicating that its population structure was sustained by keystone core species (Supplementary Table 3). However, because keystone taxa act as “ecosystem engineers” within networks, exerting significant regulatory influence over network structure and function, a decline in their numbers may weaken network stability and even lead to the collapse of network structure (Banerjee et al., 2018). Dispersal and physical barriers can serve as additional factors affecting community assemblages (Xie et al., 2017). Compared to mono-culture, the lotus-fish co-culture system may enhance physical disturbance of the sediments, limiting the formation of high-density interaction networks and resulting in a weaker network structure. We additionally observed that the indices Eff, Eigen and Pcr of sediment and water column bacterial networks in LP declined much faster when strong edges and strong nodes were deleted (Figures 6D–G). Nevertheless, in terms of network vulnerability, the bacterial networks in both the sediment and water column of LP showed lower vulnerability, while those of MP displayed higher values (Figure 6H). According to three statistical metrics, LP presents weak interspecific dependence, where removal driven by random perturbations rarely causes cascading effects and leads to low holistic vulnerability. The compact MP mutualistic network with high positive cohesion is robust against core species elimination, yet dense beneficial interactions accelerate disturbance transmission, predisposing MP communities to deterioration under stochastic interference.

RDA analysis revealed that sediment bacterial communities in MP were more strongly structured by nutrients relative to LP, indicating MP microbes were subjected to greater nutrient stress (Figure 7A). Consistent with this, WGCNA module-environment correlation identified TN as the dominant positive driver in MP (Figure 7C). By contrast, LP harbored more modules with positive or negative correlations to TC, TN and TP, and its core modules were closely associated with coupled carbon, nitrogen and phosphorus cycling (Figure 7B). Previous studies have shown that keystone with wider niche breadths are less influenced by environmental factors, whereas specialists with narrower niche breadths are more affected (Logares et al., 2020). Due to keystone’s higher functional diversity, this group typically exhibits broader niche widths and stronger environmental adaptability. Accordingly, they can accelerate organic matter decomposition and nutrient transformation to boost the bioavailability of inorganic salts within the system, further improving the utilization efficiency of other peripheral species. In MP sediments, total nitrogen (TN) acts as the primary regulator enriching bacterial taxa, while total phosphorus (TP) exerts negative effects. This pattern can be attributed to abundant inorganic nitrogen, which supplies sufficient respiratory substrates for numerous bacterial groups under low-oxygen conditions. Meanwhile, the declining TP concentration in MP may intensify phosphorus competition among microorganisms. The number of modules positively or negatively correlated with all nutrients was markedly higher in LP than MP. Lotus plants supply diverse carbon sources, and frequent sediment disturbance creates gradient microhabitats, driving bacterial populations to differentiate into groups with divergent nutrient preferences. Accordingly, TC, TN and TP all filter a large number of taxa with positive or negative nutrient responses. LP contained abundant carbon-associated modules because lotus residues provide varied organic carbon, making carbon a key selective factor. In contrast, MP had far fewer carbon-related modules due to its single carbon source, and total carbon exerted weak regulatory effects on bacterial community assembly.

At 1 year of age, the FBW, FBL and CF of fish in the LP ponds remained significantly greater than those in the MP ponds (Table 1). Throughout the whole experimental period, no significant differences were observed in WGR and specific growth rate (SGR) between the two treatments. Although no significant difference was observed in fish growth rate, which may be mainly determined by feed ration and fish feeding intensity, fish in LP ponds achieved significantly higher final body weight and body length than those in MP ponds, with lower condition factor and more streamlined body shape. Lotus plants supply abundant natural food resources including insects, zooplankton and plant detritus. Their photosynthesis helps regulate dissolved oxygen in the pond, and transpiration moderates water temperature. Furthermore, lotus cultivation reshapes sediment microecology to facilitate fish growth. Diverse organic matter derived from lotus drives the differentiation of bacterial modules responsible for integrated carbon, nitrogen and phosphorus cycling. These microbial communities efficiently decompose residual feed and plant/animal debris. Meanwhile, nitrogen-removing bacteria in sediment reduce toxic nitrogen compounds such as ammonia and nitrite, optimize sediment oxygen conditions, and alleviate fish stress and disease risks.

## Conclusion

Lotus vegetation provides habitats and breeding grounds for insects and zooplankton, and forms a natural food pool together with plant detritus to supplement artificial feed. Compared with mono-fish ponds, fish cultured under lotus-fish co-culture obtain higher final body weight and length, with more streamlined body shape and better condition factor. Lotus plants directly absorb nitrogen and phosphorus from water to alleviate excessive phosphorus accumulation in sediment, avoiding microbial community imbalance caused by high phosphorus stress in mono-culture and sustaining a stable aquatic environment suitable for fish survival. Furthermore, diverse organic carbon supplied by lotus residues drives sediment bacteria to differentiate into functional modules responsible for coupled carbon, nitrogen and phosphorus cycling, which efficiently degrade residual feed and organic debris. Nitrogen-removal microbial communities reduce toxic nitrogen compounds such as ammonia and nitrite, ameliorate hypoxic sediment conditions, relieve fish stress and lower the incidence of diseases. Diversified carbon sources select microbial groups with distinct nutrient preferences and clear functional division. The microbial community exhibits strong buffering capacity against nutrient fluctuations to stabilize water quality, thereby reducing the risks of growth retardation and mass disease outbreaks in fish from the perspective of underlying sediment microecology.

## Data Availability

The datasets presented in this study can be found in online repositories. The names of the repository/repositories and accession number(s) can be found in the article/Supplementary material.

## Footnotes

1
https://cloud.majorbio.com/page/project/overview

2 http://www.arb-silva.de with a confidence level of 70% (Wang et al., 2007).

## Supplementary material

Supplementary Figure 1 The AWCD (Average Well Color Development, 590nm) of carbon source metabolism in the microbial of sediment. LM = Sediment samples from lotus ponds (LP); MM = Sediment samples from monoculture ponds (MP).

Supplementary Figure 2 Line graph displays the concentration of chlorophyll *a* in both pond groups. LW = Water samples from lotus ponds (LP); MW = Water samples from monoculture ponds (MP).

Supplementary Figure 3 Bacterial community composition of top10 genus. LM = Sediment samples from lotus ponds (LP); LW = Water samples from lotus ponds (LP); MM = Sediment samples from monoculture ponds (MP); MW = Water samples from monoculture ponds (MP).
