# Supplementary material for: Lotus-fish co-culture reshapes pond microbiota and improves ecological stability relative to fish monoculture
Source: Front Microbiol. 2026 Aug 26;17:1922152. doi: 10.3389/fmicb.2026.1922152 (PMC13553798; doi:10.3389/fmicb.2026.1922152)
Supplement: Supplementary file 1 [file Supplementary_file_1.docx]

**Total Nitrogen (TN) in Sediment**

**Experimental Principle**
Most nitrogen in soil exists in organic forms (proteins, amino acids, humus, amides, etc.), while inorganic nitrogen (NH₄⁺, NO₃⁻, NO₂⁻) accounts for a very small proportion. The total nitrogen content is determined by the humus content of soil. Under the action of reducing catalysts, nitrogen-containing organic compounds in soil are digested and decomposed with sulfuric acid. The ammonia released from decomposition reacts with sulfuric acid to form ammonium sulfate. Subsequently, ammonia is liberated via alkalization and distillation, absorbed by boric acid, and titrated with standard hydrochloric acid solution using methyl red-bromocresol green as the indicator until the solution turns pink as the endpoint. The total nitrogen content in soil is calculated based on the consumption of hydrochloric acid.

**Reagents**

(a) Boric acid receiving solution (10 g/L): Dissolve 100 mg bromocresol green in 100 mL ethanol to prepare 0.1% bromocresol green solution. Separately dissolve 100 mg methyl red in 100 mL ethanol to prepare 0.1% methyl red solution. Weigh 100 g boric acid, dissolve with deionized water and make up to 10 L volumetric volume. Add 100 mL 0.1% bromocresol green solution and 70 mL 0.1% methyl red solution to obtain the 10 g/L boric acid receiving solution.

(b) Standard volumetric sulfuric acid solution: 0.02 mol/L. Note: Corrected in translation according to the final calculation formula typo in original text

(c) Mixed catalyst: Uniformly mix and grind 100 g potassium sulfate, 10 g finely ground copper sulfate and 1 g selenium powder thoroughly.

(d) Mixed indicator: Dissolve 0.5 g bromocresol green and 0.1 g methyl red in 100 mL 95% ethanol. Adjust the solution to pale purple with dilute sodium hydroxide or dilute hydrochloric acid, with a final pH of 4.5. **Do not heat during preparation; store under refrigeration.**

**Experimental Procedures**

**1. Digestion**

Accurately weigh approximately 0.5000 g air-dried soil sample passed through a 0.25 mm (or 0.5 mm) sieve, add 1.8 g mixed catalyst and 5 mL concentrated sulfuric acid. Place the digestion tube on a digestion furnace inside a fume hood and digest at 420 °C for 2 hours until the solution becomes clear light blue.

**2. Distillation**

Place an Erlenmeyer flask containing 20 mL 2% boric acid solution under the delivery tube of the condenser, ensuring the tube tip is submerged in the boric acid solution. Turn on cooling water and start distillation. Continue distilling for another 5 minutes after the receiving solution turns blue. Rinse the outer wall of the delivery tube with distilled water upon completion.

**3. Titration**

Titrate the receiving solution with 0.02 mol/L standard hydrochloric acid solution until the solution turns red, and record the consumed volume of standard hydrochloric acid.

**4. Blank Control**

All procedures are identical except that no soil sample is added.

**Sample Test Result Calculation**

The total nitrogen content in soil is calculated by the following formula:

$$(Soil nitrogen content (mg/g)=\frac{(V-V_{0})\times N\times0.014}{W})$$

**Parameter definition:**

V: Volume of standard hydrochloric acid consumed for sample titration (mL)

V0: Volume of standard hydrochloric acid consumed for blank titration (mL)

N: Molar concentration of standard hydrochloric acid titrant, 0.01 mol/L

W: Mass of soil sample (g)

0.014: Milliequivalent weight of nitrogen

**Total Carbon (TC) in Sediment**

**Experimental Principle**

The entire testing procedure for sediment total carbon (TC) consists of six steps: sample collection, grinding and sieving, sample pretreatment, instrumental analysis, data processing and quality control, and report issuance. After sample combustion, the products are delivered to the detector of an elemental analyzer, and the carbon content of the sample is calculated by comparison with certified reference materials.

Spectroscopic instruments quantify target compounds based on the characteristic absorption peaks of substances at specific wavelengths. Analytes are generally categorized into two types:

1.Direct analytes: Target substances themselves have characteristic absorption peaks at specific wavelengths and can be directly quantified.

2.Indirect analytes: Quantification relies on chemical or physical reactions; the concentration of the original target compound is determined by measuring the characteristic absorption peaks of reaction products.

This test also determines total phosphorus content in sediment. Soil samples are fused with sodium hydroxide to convert phosphorus-bearing minerals and organic phosphorus compounds into soluble orthophosphates. Under acidic conditions, orthophosphates react with molybdenum-antimony-ascorbic acid chromogenic reagent to form phosphomolybdenum blue, whose absorbance is measured at a wavelength of 700 nm.

**Reference Standard and Reagents**

δ13CV-PDB [δ13C, -13.78±1.7‰]is adopted as the reference material.

**Experimental Procedures**

An appropriate amount of test sample is weighed and encapsulated in a tin capsule, then combusted in a combustion tube at 1000 °C (filled with silver wire, copper oxide and chromium oxide). The generated gas passes through a reduction tube packed with granular copper at 600 °C for reduction. After dehydration, the gas components are separated by a chromatographic column maintained at 60 °C and subsequently introduced into the detector of the elemental analyzer. The total carbon content is calculated via calibration against standard reference materials.

**Instrument Parameters**

Target analytes are quantitatively determined using an Elemental Analyzer (Sercon Integra 2, UK).

Rated operating voltage: 230 V

Ambient temperature: controlled at 22 °C

Carrier gas: high-purity helium (purity ≥ 99.99%)

Combustion-supporting gas: high-purity oxygen (purity ≥ 99.99%)

| **Equipment Name** | **Manufacturer** | **Model** |
| --- | --- | --- |
| Analytical Balance | Shenyu Longteng | ESJ30-5A |
| Elemental Analyzer | SerCon | Integra 2 |
| Ultrapure Water System | Moore | — |

**Total Phosphorus (TP) in Sediment
Experimental Principle**

The complete testing workflow includes six procedures: sample collection, grinding and sieving, sample pretreatment, instrumental analysis, data processing and quality control, and test report issuance. Sediment samples are fused with sodium hydroxide to convert phosphorus-bearing minerals and organic phosphorus compounds entirely into soluble orthophosphates. Under acidic conditions, orthophosphates react with molybdenum-antimony-ascorbic acid chromogenic reagent to form phosphomolybdenum blue, and the absorbance is measured at a wavelength of 700 nm.

**Reference Standards and Reagents**

| **Name** | **CAS No.** | **Manufacturer** |
| --- | --- | --- |
| Potassium Phosphate Monobasic | 7778-77-0 | ANPEL, China |
| Sulfuric acid | 7664-93-9 | SINOPHARM, China |
| Hydrogen peroxide | 7722-84-1 | SINOPHARM, China |
| Sodium hydroxide | 1310-73-2 | SINOPHARM, China |
| Ascorbic Acid | 50-81-7 | SIGMA |
| 2,4-dinitrophenol | 51-28-5 | SINOPHARM, China |
| Ammonium molybdate | 13106-76-8 | SINOPHARM, China |
| Potassium antimony tartrate | 16039-64-8 | SINOPHARM, China |

**(a) 2,4-Dinitrophenol Indicator**

Weigh 0.2 g of 2,4-dinitrophenol and dissolve in 100 mL of deionized water.

(b) Molybdenum-Antimony-Ascorbic Acid Chromogenic Reagent

Dissolve 10 g ammonium molybdate in 300 mL warm water (~60 °C) and cool to room temperature. Slowly pour 126 mL concentrated sulfuric acid into approximately 400 mL water under continuous stirring, then cool down. Gradually add the diluted sulfuric acid into the ammonium molybdate solution and mix thoroughly. Add 100 mL of 5 g/L potassium antimony tartrate solution, make up to 1000 mL with water, and store away from light. Before use, dissolve 1.5 g ascorbic acid into the mixed solution. This reagent should be freshly prepared before each use.

(c) Preparation of Phosphorus Standard Solutions

1.**Standard stock solution**: Accurately weigh 0.4390 g potassium dihydrogen phosphate dried at 105 °C for 2 h, dissolve in water, add 5 mL concentrated sulfuric acid, cool and dilute to 1000 mL with water to obtain phosphorus standard stock solution.

2.**Working standard solution**: Pipette 5 mL of the stock solution into a 100 mL volumetric flask and dilute to volume with water to prepare 5 mg/L phosphorus working standard solution. This working solution is not suitable for long-term storage.

**Sample Pretreatment**

**1. Grinding and Homogenization**

Spread collected sediment samples thinly (2–3 cm thickness) on an air-drying tray for air drying. Crush and turn over the samples periodically, picking out gravel, stone fragments and plant residues. Grind the air-dried samples with a wooden pestle, remove impurities, fully homogenize and pass through a 1 mm soil sieve. Spread the sieved samples thinly on kraft paper and divide into small grids using the quartering method. Collect equal amounts of sediment from each grid with a small spoon (total weight >20 g), further grind the subsamples with a soil grinder or ball mill until all particles pass through a 0.149 mm (100-mesh) sieve. Mix uniformly and store in a ground-glass stoppered bottle for later analysis.

**2. Digestion by Alkali Fusion**

Accurately weigh 0.2 g sample into the bottom of a nickel crucible and moisten with a few drops of absolute ethanol. Spread 2 g sodium hydroxide evenly on the sample surface to fully cover the sample, then cover the crucible with a lid. Place the crucible in a muffle furnace: heat up to around 400 °C and hold for 15 min, then continuously raise the temperature to 640 °C and maintain for another 15 min. Take out the crucible and cool to ambient temperature. Add 10 mL water into the crucible and heat to 80 °C to dissolve the fused block. Transfer all liquid into a 50 mL centrifuge cup. Rinse the crucible three times with 10 mL sulfuric acid solution in batches and combine all washing liquids into the centrifuge cup, followed by three additional rinses with an appropriate volume of water. Centrifuge the mixture at 2500–3500 r/min for 10 min. Transfer all supernatant into a 100 mL volumetric flask and dilute to the mark with water for determination.

**3. Colorimetric Determination**

Pipette 1 mL of the digested supernatant into a 50 mL volumetric flask, add water to about 30 mL, drop 2 drops of 2,4-dinitrophenol indicator, and adjust pH until the solution turns faint yellow. Add 5 mL molybdenum-antimony-ascorbic acid chromogenic reagent and make up to volume with water. Allow color development for 30 min at temperature above 20 °C. Measure absorbance at 700 nm on a spectrophotometer using a 1 cm optical path cuvette, with the zero point of the standard curve set as the blank reference.

**4. Blank Control**

All experimental steps are identical except that no sediment sample is added.

**5. Standard Curve Establishment**

Accurately pipette 0 mL, 1 mL, 2 mL, 3 mL, 4 mL, 5 mL and 6 mL phosphorus working standard solution into separate 50 mL volumetric flasks respectively. Add the same volume of blank digestion solution as used for sample testing, fill with water to approximately 30 mL, add 2 drops of 2,4-dinitrophenol indicator and adjust pH to faint yellow. Add 5 mL chromogenic reagent and dilute to volume. The corresponding phosphorus masses in these solutions are 0 μg, 5 μg, 10 μg, 15 μg, 20 μg, 25 μg and 30 μg. Measure absorbance values and establish the linear standard curve.

**Instrument Parameters**

A temperature-controlled far-infrared digestion furnace (220 V±10%, 50 Hz, 2000 W) was used for sample digestion. Quantitative detection of total phosphorus was performed on a Thermo Fisher (USA) ultraviolet-visible spectrophotometer, model AquaMate 8100.

**Calculation of Sample Total Phosphorus Content**

$$(Total Phosphorus in sample (mg/kg)=\frac{C\times(V\div V_{0})}{W})$$

C: Phosphorus concentration calculated from the standard curve

V: Volume of solution taken for color measurement (mL)

V0: Final constant volume of digested extract (mL)

W: Weight of sediment sample (g)

0.001: Unit conversion factor

**Instrument List**

| **Equipment Name** | **Manufacturer** | **Model** | **Instrument No.** |
| --- | --- | --- | --- |
| Analytical Balance | Shenyu Longteng | ESJ30-5A | — |
| Temperature-controlled Far-infrared Digestion Furnace | Jiangsu Shenglan | LWY-84B | — |
| UV-Vis Spectrophotometer | ThermoFisher | AquaMate 8100 | — |
| Ultrapure Water System | Moore | — | — |
| Muffle Furnace | Shanghai Techeng | SX-4-10 | — |

**Organic Matter (OM) in Sediment**

**1.Experimental Principle**
The full analytical workflow for sediment organic matter (OM) consists of six steps: sample collection, crushing and sieving, sample pretreatment, instrumental measurement, data processing and quality control, and final report issuance. This method quantifies soil organic carbon (SOC) content.

Under heating conditions, excessive potassium dichromate-sulfuric acid solution oxidizes organic carbon in sediments. Residual unreacted dichromate is titrated with standard ammonium ferrous sulfate solution. Organic carbon content is calculated based on the volume difference of titrant consumed between sample and blank tests.

This wet oxidation method only oxidizes approximately 90% of total organic carbon compared with the dry combustion method. Therefore, the measured organic carbon value is multiplied by a correction factor of 1.1 to obtain the corrected total organic carbon content. Subsequently, the Van Bemmelen factor of 1.724 is applied to convert organic carbon to total organic matter content.

**2.Reference Standards and Reagents**

| **Name** | **CAS No.** | **Manufacturer** |
| --- | --- | --- |
| Potassium dichromate | 7778-50-9 | SINOPHARM, China |
| Ferrous sulfate heptahydrate | 7782-63-0 | SINOPHARM, China |
| Concentrated sulfuric acid | 7664-93-9 | SINOPHARM, China |
| 1,10-Phenanthroline hydrate | 5144-89-8 | ANPEL, China |
| Silver sulfate | 10294-26-5 | SIGMA |

**1,10-Phenanthroline Indicator**

Dissolve 0.695 g pure ferrous sulfate and 1.485 g analytical-grade 1,10-phenanthroline in 100 mL deionized water. The reagent forms a reddish-brown complex [Fe(C_12_H_8_N_2_)_3_}]^2+^with ferrous ions.

**3. Sample Pretreatment & Determination Procedures**

**3.1 Weighing and Oxidation Digestion**

**1.**Accurately weigh 0.5000 g air-dried sediment passing through a 0.25 mm sieve into a 20 mL hard glass test tube via the weighing-by-difference method on an analytical balance.

**2.**Add 0.1 g silver sulfate, pipette 5 mL 0.8 mol/L potassium dichromate standard solution, followed by 5 mL concentrated sulfuric acid, and shake gently for thorough mixing. A small amount of silica powder can be added to prevent bumping during boiling.

**3.**Preheat the paraffin oil bath to 185–190 °C. Place test tubes in an iron cage and immerse the cage into the oil bath. Maintain the bath temperature at 170–180 °C, keep the solution boiling continuously for 5 min, then take out and cool to room temperature

**3.2 Titration
1.**Transfer all digested mixture from the test tube into a 250 mL Erlenmeyer flask, and adjust the final volume to 60–80 mL with distilled water.

**2.**Add 3–5 drops of 1,10-phenanthroline indicator, titrate with 0.2 mol/L ammonium ferrous sulfate standard solution until the color sharply changes from yellow through green to reddish-brown (titration endpoint). Record the consumed volume of titrant.

**3.**Three parallel blank tests using quartz sand instead of sediment samples are run simultaneously for each batch, and the average blank volume is adopted for calculation.

**4. Calculation Formula for Organic Matter Content**

$$(Organic Matter (g/kg)=\frac{N\times(V_{0}-V)\times0.003\times1.1\times1.724\times1000}{W})$$

**Parameter Explanation**

N: Molar concentration of ammonium ferrous sulfate standard solution (mol/L)

V0: Volume of titrant consumed in blank test (mL)

V: Volume of titrant consumed in sample test (mL)

0.003: Milliequivalent mass of carbon (g)

1.1: Oxidation efficiency correction factor for wet digestion method

1.724: Van Bemmelen conversion factor (organic carbon to organic matter, assuming OM contains 58% carbon)

1000: Unit conversion factor (convert g/g to g/kg)

W: Weight of air-dried sediment sample (g) **5. Instrument List**

| **Equipment Name** | **Manufacturer** | **Model** | **Instrument No.** |
| --- | --- | --- | --- |
| Analytical Balance | Shenyu Longteng | ESJ30-5A | — |
| Electric Drying Oven | Yiheng | DHG-9240A | Shenzhen, China |
| Thermostatic Oil Bath | Yiheng | DU-20/30 | Shenzhen, China |
| Semimicro Burette | — | — | — |
