# Supplementary material for: Lotus-fish co-culture reshapes pond microbiota and improves ecological stability relative to fish monoculture
Source: Front Microbiol. 2026 Aug 26;17:1922152. doi: 10.3389/fmicb.2026.1922152 (PMC13553798; doi:10.3389/fmicb.2026.1922152)
Supplement: Supplementary file 2 [file Table_1.docx]

Supplement Table 1 Results of PERMANOVA analysis for factors structuring sediment bacterial communities

| Name | SumsOfSqs | MeanSqs | F.Models | R^2^ | Pr(>F) |
| --- | --- | --- | --- | --- | --- |
| C | 0.51 | 0.51 | 5.29 | 0.13 | 0.001 |
| N | 0.53 | 0.53 | 5.55 | 0.14 | 0.001 |
| OM | 0.51 | 0.51 | 5.29 | 0.13 | 0.001 |
| P | 0.47 | 0.47 | 4.80 | 0.12 | 0.002 |
| Pond | 0.84 | 0.84 | 9.71 | 0.22 | 0.001 |
| month | 0.22 | 0.22 | 2.06 | 0.08 | 0.042 |

C: Total carbon;
N: Total nitrogen;
OM: Organic matter;

P: Total phosphorus;

Pond: Aquaculture culture mode(LP/MP);

month: Sampling month;
SumsOfSqs: Sum of squares;
MeanSqs: Mean square;
F.Models: F-statistic of the model;
R²: Proportion of community variation explained by each factor;
Pr (>F): Significance value of F-test. All significant P-values were set at α = 0.05.
