# Supplementary material for: Lotus-fish co-culture reshapes pond microbiota and improves ecological stability relative to fish monoculture
Source: Front Microbiol. 2026 Aug 26;17:1922152. doi: 10.3389/fmicb.2026.1922152 (PMC13553798; doi:10.3389/fmicb.2026.1922152)
Supplement: Supplementary file 3 [file Table_2.docx]

Supplement Table 2 Results of PERMANOVA analysis for factors structuring water bacterial communities

| Name | SumsOfSqs | MeanSqs | F.Models | R^2^ | Pr(>F) |
| --- | --- | --- | --- | --- | --- |
| Pond | 1.77 | 1.77 | 8.25 | 0.20 | 0.001 |
| month | 1.50 | 1.52 | 6.76 | 0.17 | 0.001 |

Pond: Aquaculture culture mode(LP/MP);

month: Sampling month;
SumsOfSqs: Sum of squares;
MeanSqs: Mean square;
F.Models: F-statistic of the model;
R²: Proportion of community variation explained by each factor;
Pr (>F): Significance value of F-test. All significant P-values were set at α = 0.05.
