# Supplementary material for: Lotus-fish co-culture reshapes pond microbiota and improves ecological stability relative to fish monoculture
Source: Front Microbiol. 2026 Aug 26;17:1922152. doi: 10.3389/fmicb.2026.1922152 (PMC13553798; doi:10.3389/fmicb.2026.1922152)
Supplement: Supplementary file 4 [file Table_3.docx]

Supplement Table 3Major topological properties of co-occurrence network of bacterial communities in water and sediment with different aquaculture ponds

| Group | Nodes | Edges | Modularity | Average clustering  coefficient | Average path  length | Average  degree |
| --- | --- | --- | --- | --- | --- | --- |
| LM | 1071 | 6752 | 0.451 | 0.314 | 4.410 | 6.304 |
| MM | 1133 | 42021 | 0.372 | 0.492 | 2.855 | 74.177 |
| LW | 550 | 14870 | 0.354 | 0.570 | 2.811 | 54.073 |
| MW | 503 | 13770 | 0.321 | 0.570 | 2.774 | 54.751 |
