# Supplementary material for: Lotus-fish co-culture reshapes pond microbiota and improves ecological stability relative to fish monoculture
Source: Front Microbiol. 2026 Aug 26;17:1922152. doi: 10.3389/fmicb.2026.1922152 (PMC13553798; doi:10.3389/fmicb.2026.1922152)
Supplement: Supplementary file 5 [file Table_4.docx]

Supplement Table 4 keystone taxa in bacterial co-occurrence networks

| Group | Peripherals | Connectors | Module hubs | Network hubs |
| --- | --- | --- | --- | --- |
| LM | 1007 | 36 | 26 | 2 |
| MM | 1095 | 28 | 10 | / |
| LW | 529 | 12 | 9 | / |
| MW | 478 | 10 | 15 | / |
